# Leveraging model-informed approaches for drug discovery and development in the cardiovascular space

**DOI:** 10.1007/s10928-018-9571-3

**Published:** 2018-01-20

**Authors:** Marissa F. Dockendorf, Ryan C. Vargo, Ferdous Gheyas, Anne S. Y. Chain, Manash S. Chatterjee, Larissa A. Wenning

**Affiliations:** Pharmacokinetics, Pharmacodynamics, and Drug Metabolism, Merck & Co., Inc., Kenilworth, NJ USA

**Keywords:** Cardiovascular, Pharmacokinetic/pharmacodynamic modeling, Exposure–response, Drug development, Clinical trial simulation

## Abstract

Cardiovascular disease remains a significant global health burden, and development of cardiovascular drugs in the current regulatory environment often demands large and expensive cardiovascular outcome trials. Thus, the use of quantitative pharmacometric approaches which can help enable early Go/No Go decision making, ensure appropriate dose selection, and increase the likelihood of successful clinical trials, have become increasingly important to help reduce the risk of failed cardiovascular outcomes studies. In addition, cardiovascular safety is an important consideration for many drug development programs, whether or not the drug is designed to treat cardiovascular disease; modeling and simulation approaches also have utility in assessing risk in this area. Herein, examples of modeling and simulation applied at various stages of drug development, spanning from the discovery stage through late-stage clinical development, for cardiovascular programs are presented. Examples of how modeling approaches have been utilized in early development programs across various therapeutic areas to help inform strategies to mitigate the risk of cardiovascular-related adverse events, such as QTc prolongation and changes in blood pressure, are also presented. These examples demonstrate how more informed drug development decisions can be enabled by modeling and simulation approaches in the cardiovascular area.

## Introduction

Cardiovascular disease represents a significant global health burden. It remains the primary global cause of death, leading to approximately 17.3 million deaths in 2013, and estimated direct and indirect costs of over $316 billion in 2012–2013, continuing to rise [1]. Development of drugs to treat cardiovascular disease is challenging, and in the current regulatory environment often requires the conduct of large and expensive outcomes trials. Increasing attention has been given in recent years to the role of quantitative modeling and simulation tools to enable early Go/No Go decision making, ensure appropriate dose selection, and increase the likelihood of successful clinical trials [2]. Consistent use of model-informed drug discovery and development approaches will ideally shift discontinuation of non-viable compounds earlier in development, resulting in more time- and resource-efficient drug development paths and reducing the risk of failed cardiovascular outcomes studies. Furthermore, for compounds that do proceed to outcome trials, it is likely that only one dose can be studied given the high cost of such trials, and modeling and simulation approaches can be utilized to select the optimal Phase 3 dose. Cardiovascular safety is an important consideration for many drug development programs, whether or not the drug is designed to treat cardiovascular disease; modeling and simulation approaches also have utility in assessing risk in this area. Herein, examples of modeling and simulation applied at various stages of drug development for cardiovascular programs are described. The examples discussed span from the discovery stage through late-stage clinical development and demonstrate how pharmacometric model-based approaches can be applied throughout drug development to enable more informed development decisions for cardiovascular programs. Additional examples of how modeling and simulation approaches can be used in early phase development across therapeutic areas in order to inform strategies to mitigate the risk of cardiovascular-related adverse events are also presented.

## Cardiovascular drug discovery

In the discovery phase of drug development, compounds are screened in various in vitro and animal studies. At this stage, quantitative and translational approaches may be applied to help prioritize compounds and targets that are differentiated from currently available treatments. Quantitative tools applied at this stage can be used to project pharmacokinetic behavior in humans, to characterize dose- and/or exposure–response (ER) relationships in preclinical systems and translate to the likely therapeutic window in humans, and to predict drug–drug interactions [3–5]. Furthermore, assessment of the competitive landscape and benchmarking to other therapies through the use of comparator modeling can be a valuable tool to help identify appropriate targets for safety and efficacy for new therapies prior to first-in-human studies. Together, these quantitative approaches allow selection of improved candidates for clinical assessment and design of a rational and efficient clinical development program. An example of how translational pharmacokinetic/pharmacodynamic (PK/PD) modeling approaches were used in a cardiovascular discovery program in order to define the possible therapeutic window for compounds with a new mechanism of action (Factor IXa [f1Xa] inhibition) relative to approved therapies is presented to illustrate some of these principles.

### Example: Informing therapeutic window in the discovery phase

Novel oral anticoagulants that block coagulation factor Xa (fXa), such as apixaban, have been used for the prevention of stroke and systemic embolism in patients with atrial fibrillation (SPAF); however, despite their effectiveness, these therapies have high incidences of major and nonmajor clinically relevant bleeding (~ 15% for atrial fibrillation patients) [6–8]. Genetic evidence suggests that reduced fIXa activity can confer protection against thrombosis [9, 10], and it was hypothesized that since fIXa lies upstream of fXa in the coagulation cascade, fIXa inhibition may have decreased risk of bleeding as compared to fXa inhibitors. Therefore, Ankrom et al. [11] evaluated whether fIXa inhibitors could provide an improved therapeutic window vs. fXa inhibitors in the preclinical/discovery phase of fIXa inhibitor drug development. They studied the efficacy and safety of a fIXa inhibitor, CPD1, relative to the fXa inhibitor, apixaban, in rats, and used translational PK/PD model-based approaches to evaluate these data and support drug development decisions for the discovery program.

**Fig. 1:**
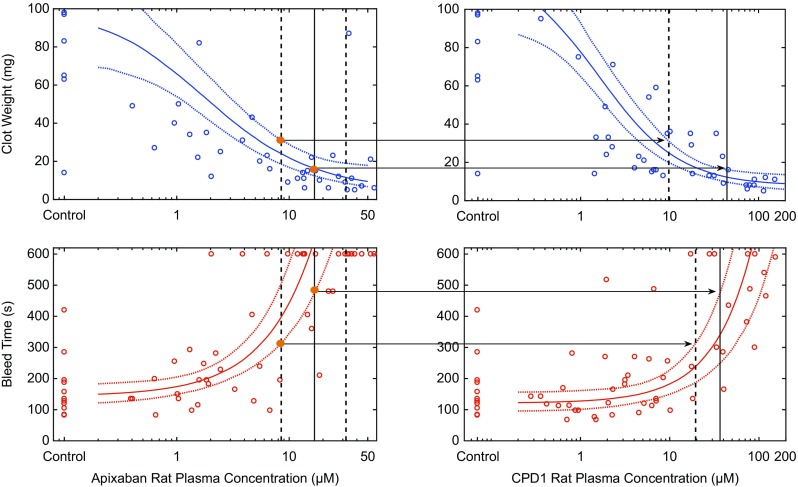
Translational pharmacokinetics/pharmacodynamics (PK/PD) analysis of the efficacy/bleeding study. PK/PD model fits (solid line: median; dotted lines: 5th and 95th percentiles incorporating uncertainty of parameter estimates) are overlaid with observed (circles) clot weight (top panels) and bleed time (bottom panels) as a function of apixaban (left panels) and compound 1 (CPD1) (right panels) rat plasma concentrations. The vertical lines on the apixaban figures represent the median (solid) and 90% CI (dotted) range of clinically relevant apixaban C_trough_ concentrations. Vertical lines on the right panels correspond to the concentrations of CPD1 that achieve clot weight and bleed times equivalent to apixaban. Adapted with permission from Ankrom et al. [11]

In their analysis, clot weight inhibition in a rat arteriovenous shunt model and cuticle bleeding times were measured across a range of exposures for both compounds; regression models were used to quantify the shape of these exposure–response relationships (Fig. 1) [11]. Clinically relevant concentrations of apixaban in rats were defined as the concentration range expected to yield the same levels of fXa enzyme occupancy as achieved by trough concentrations (C_trough_) of a 5 mg BID dose of apixaban (approved for SPAF), after accounting for experimental uncertainty in potency and protein binding across species. The range of clot weight and cuticle bleeding time levels achieved by clinically relevant concentrations of apixaban in rats was used to establish preclinical efficacy and safety targets for CPD1 to achieve equivalent/superior therapeutic index to apixaban.

It was demonstrated that there existed a narrow range of CPD1 concentrations (> 9.8 and < 19.5 µM), corresponding to 65–79% fIXa enzyme occupancy levels, where CPD1 could exceed the level of clot weight inhibition achieved by the 5th percentile of clinically relevant rat exposure of apixaban and still have less bleeding. However, concentrations of CPD1 that achieved efficacy greater than that achieved by the median clinically relevant rat exposure of apixaban and also maintained bleeding risk below that of apixaban’s could not be identified in this analysis. The results of this analysis helped define the anticipated therapeutic window of CPD1 in humans relative to a comparator based on preclinical data and informed strategic discussion on the viability of fIXa as an antithrombotic target.

## Cardiovascular drug clinical development

In clinical development, quantitative approaches can be leveraged in a multitude of ways, including to assess therapeutic window, support trial design and dose/regimen selection, support formulation bridging, and benchmark response to marketed therapies. Approaches used in this space can range from population pharmacokinetics (PK) and empirical ER models to more complex disease and quantitative systems pharmacology models to model-based meta-analysis [2].

The impact of model-informed decision making can also extend into the post-approval stage, where the continued development of therapies may occur in order to extend product value, provide patients with more convenient dosing options (e.g., fixed-dose combinations [FDC]), and/or reduce product cost (e.g., manufacturing changes). At this stage of development, the impact of modeling and simulation can even lead to clinical trial avoidance in some cases. For example, developing in vitro-in vivo correlation (IVIVC) models that predict in vivo PK performance based on in vitro dissolution data can provide a streamlined path towards regulatory approval of post-marketing manufacturing changes without the need to conduct additional clinical studies [12, 13].

Presented below are three examples of how modeling and simulation approaches have been applied at various stages of development for cardiovascular programs in order to inform dose selection for late phase studies (anacetrapib example), inform the possible need for dose adjustment based on intrinsic and extrinsic factors and support label claims (vorapaxar example), and inform drug development for an FDC of two already marketed therapies (ezetimibe + atorvastatin FDC example).

### Example: Informing dose selection for late phase trials

Anacetrapib is a novel cholesteryl ester transfer protein (CETP) inhibitor designed for cardiovascular risk reduction and the treatment of hypercholesterolemia and mixed dyslipidemia. A large outcome trial of anacetrapib has recently been completed [14]. Quantitative strategies were leveraged throughout the development of anacetrapib, and the example discussed here relates to dose selection for anacetrapib Phase 3 studies, where Krishna et al. [15] used model-based approaches to support justification for studying a formulation and dose in Phase 3 that had previously not been studied in patients.

After the completion of the Phase 2b study, population PK and PK/PD modeling were performed in order to inform the Phase 3 dose. The population PK model was developed utilizing data from several Phase 1 studies as well as the Phase 2b trial. Most of the Phase 1 studies, as well as the Phase 2b study, had been conducted using the liquid-filled capsule (LFC) formulation. Two Phase 1 studies had explored the new hot-melt extruded (HME) tablet formulation. The population PK model accounted for differences between the LFC formulations and final market formulation (HME tablet) allowing bridging of the two formulations.

To characterize the relationship between PK and pharmacodynamics (PD) low-density lipoprotein cholesterol (LDL-C) and high-density lipoprotein cholesterol (HDL-C), nonlinear mixed effects PK/PD models were developed based on data obtained from multiple Phase 1 studies as well as the Phase 2b study. Proportional E_max_ models quantified the relationships between anacetrapib C_trough_ and lipoprotein effects (LDL-C and HDL-C), with covariate effects of study population (normal volunteers vs. patients) and coadministration with HMG-CoA reductase inhibitors (statins).

**Fig. 2:**
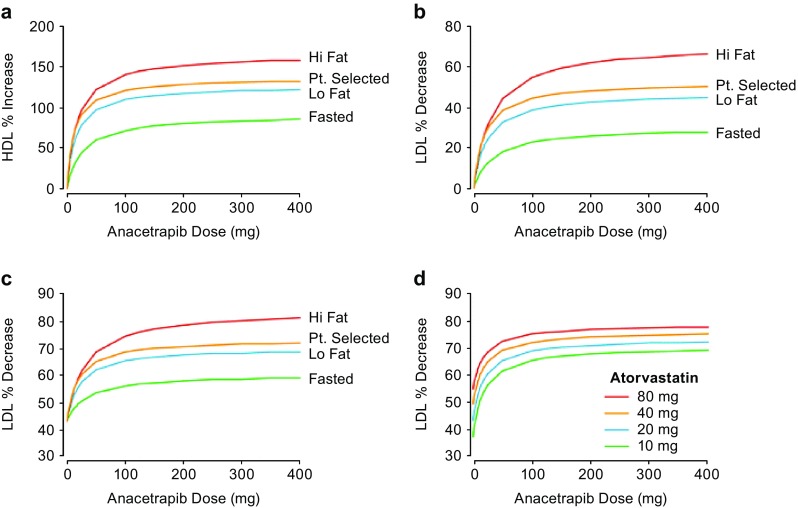
Population mean predicted HDL-C and LDL-C effects. The population mean predicted effect of fed state and meal type on HDL-C in patients treated with anacetrapib monotherapy (**a** top left). The population mean predicted effect of fed state and meal type on LDL-C in patients treated with anacetrapib monotherapy (**b** top right) or in combination with 20 mg atorvastatin (**c** bottom left). The population mean predicted effect of atorvastatin dose on LDL-C in patients treated with anacetrapib in combination with atorvastatin (**d** bottom right). *Hi Fat* standard high fat meal, *Pt. selected* patient-selected meal, *Lo Fat* standard low fat meal. Adapted with permission from Krishna et al. [15]

Clinical trial simulations were used to examine the predicted LDL-C and HDL-C effects as a function of various anacetrapib and atorvastatin doses (Fig. 2) [15], the effect of covariates and model uncertainty on the expected response, and the robustness of the effects to random dietary indiscretion. The results suggested that a 100 mg dose would result in lipid-altering effects at or near the pharmacodynamic plateau and that the predicted lipid effects were robust as long as patients generally adhered to taking their dose with a meal. Thus, a 100 mg once-daily dose with a meal using the HME formulation was selected as the Phase 3 dose. This was one of the early examples of cases at Merck & Co., Inc. (Kenilworth, NJ, USA) where modeling results facilitated the selection of a Phase 3 dose not previously studied in patient studies. This dose eventually demonstrated efficacy in six Phase 3 lipid efficacy trials as well as the cardiovascular outcome trial [14].

### Example: Informing product label

Vorapaxar is an approved protease-activated receptor-1 (PAR-1) antagonist indicated for the reduction of thrombotic cardiovascular events in patients with a history of myocardial infarction or with peripheral arterial disease. During the clinical development of vorapaxar, two of the key program questions were: (1) what is the optimal dose of vorapaxar for the majority of patients?, and (2) are there any subpopulations for which dose adjustments are needed? Population PK and PK/PD models were developed by Gheyas et al. [16] to address these questions and support regulatory submission. Vorapaxar inhibits thrombin-induced and thrombin receptor agonist peptide (TRAP)-induced platelet aggregation in in vitro studies, and vorapaxar TRAP-induced platelet aggregation (TIPA) was used as a target engagement biomarker during development and was measured in a subset of studies. A population PK/PD model was developed using TIPA as the PD endpoint and predicted concentrations from a population PK model as the PK endpoint.

**Fig. 3:**
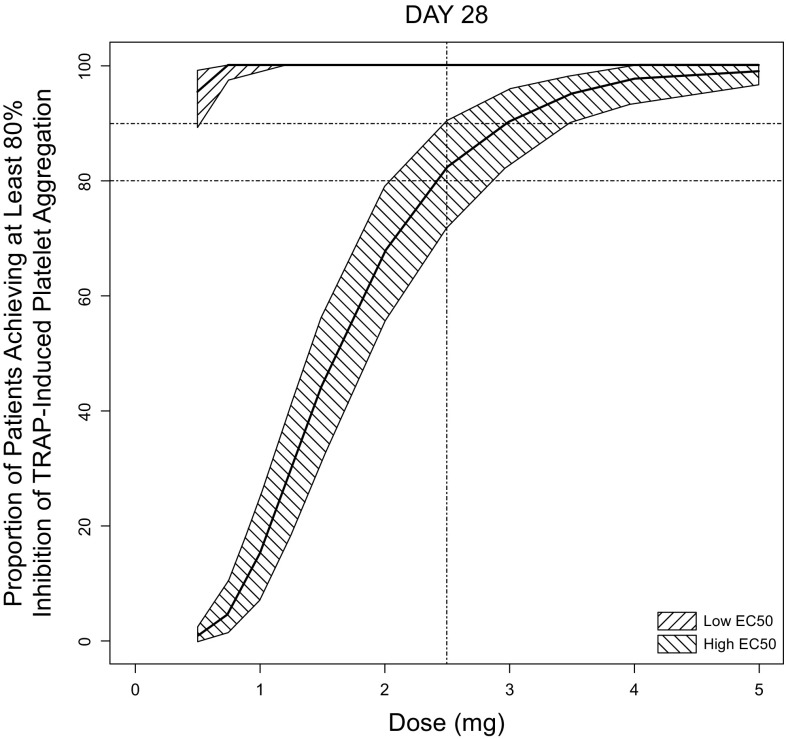
Median (95% CI) of estimated proportion of patients achieving ≥ 80% inhibition of TRAP-induced platelet aggregation based on Monte Carlo simulations utilizing the population PK and PK/PD models assuming two differing estimates of in vivo potency (EC_50_) from the modeling analysis. Reproduced with permission from Gheyas et al. [16]

The population PK model was developed using concentration–time data from dense PK sampling in 12 healthy volunteer (HV) studies and sparse PK sampling from 4 patient studies. The final population PK model was a 2-compartment model with first-order absorption. Body weight, race, gender, and creatinine clearance had mild to modest effects on vorapaxar exposure (20–40%) and were included as covariates in the population PK model. The PK/PD model to describe TIPA as a function of vorapaxar concentration was a sigmoid E_max_ model with an effect compartment. No significant covariate effects were found, except for a slight age effect (not clinically relevant, e.g., a 95-year-old patient is expected to have 9% higher EC_50_ compared to a 45-year-old patient) and a substantial study effect on EC_50_. EC_50_ was ~ 5-fold higher for two HV studies compared to that for the patient studies and the other HV studies. This difference could not be explained by demographic or study design/execution factors and was considered to be indicative of uncertainty in the PK/PD relationship. Therefore, PK/PD simulations were conducted with both values of EC_50_. The clinical pharmacodynamic target for the prevention of thrombotic events was considered to be ≥ 80% inhibition in TIPA response. Simulations based on PK and PK/PD models demonstrated that a vorapaxar sulfate dose of 2.5 mg once daily achieves ≥ 80% TIPA inhibition in most patients (Fig. 3) [16]. Simulation results also suggested that no dose adjustment based on intrinsic factors is needed. Thus, a daily dose of 2.5 mg vorapaxar sulfate was recommended in the product label for all patients who are eligible to take vorapaxar. These modeling and simulation results were included in the regulatory filing to support justification of the recommended dose and rationale for no dose adjustment for intrinsic factors.

### Example: Informing FDC development

During the development of the FDC of ezetimibe and atorvastatin, bioequivalence trials were conducted across a range of dose combinations (10/10, 10/20, 10/40 and 10/80 mg of ezetimibe/atorvastatin). In these studies, the plasma exposure (area under the curve [AUC]) and peak plasma concentration (C_max_) of atorvastatin and unconjugated ezetimibe for the FDC were compared to those from coadministration of the marketed drugs. The results demonstrated that traditional bioequivalence bounds (90% confidence interval within [0.8, 1.25]) were met for unconjugated ezetimibe and atorvastatin AUC and C_max_ for all dose combinations except for atorvastatin C_max_ at two intermediate doses (10/20 and 10/40 mg ezetimibe/atorvastatin), for which the true geometric mean ratio of the FDC/coadministration atorvastatin C_max_ fell below 0.8. Vargo et al. [17] used a model-based meta-analysis (MBMA) to assess the clinical significance of this reduction in atorvastatin C_max_ for the ezetimibe + atorvastatin FDC as compared to ezetimibe and atorvastatin coadministration.

In this analysis, an MBMA of LDL-C-lowering for statin reducing drugs was updated from a previous dose–response model by Mandema et al. [18] using published clinical data from 245 statin trials in 106,808 patients. Additionally, linear regression models were developed to describe atorvastatin AUC and C_max_ as a function of dose, using data from the coadministration arms of the bioequivalence trials. To translate the exposure differences between marketed atorvastatin tablets coadministered with marketed ezetimibe tablets and atorvastatin in the FDC, an effective dose value was calculated, which reflected a reduced dose associated with the observed reduced exposure in the bioequivalence (BE) trial. This reduced dose was then used to predict the reduced LDL-C lowering for the FDC via the dose–response model.

**Fig. 4:**
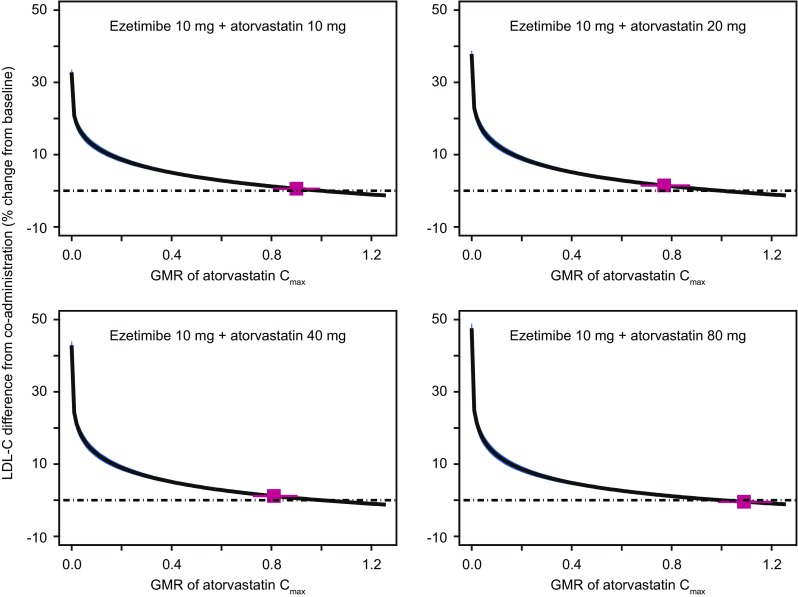
Predicted difference in low-density-lipoprotein cholesterol (LDL-C) from coadministration for ezetimibe/atorvastatin fixed-dose combination (FDC) tablets as a function of atorvastatin geometric mean ratio (GMR). Median prediction: solid line; 95% confidence interval: shaded region; GMR of the observed bioequivalence (BE) data and 90% confidence interval: boxes with lines. Reproduced with permission from Vargo et al. [17]

Combining the dose–response model with the dose-exposure model predicted that the observed difference in atorvastatin C_max_ between an ezetimibe + atorvastatin FDC and coadministration of the individual components would not translate to clinically significant changes in LDL-C (< 1.2% absolute difference in the percentage lowering of LDL-C were predicted) (Fig. 4) [17]. These analysis results were leveraged in regulatory interactions to support approval of the ezetimibe + atorvastatin FDC. Additionally, the results were used to optimally design subsequent clinical equivalence trials for the doses that did not meet BE (10/20 and 10/40 mg ezetimibe/atorvastatin) with the appropriate number of subjects based on the predicted effect size and variability across trials. The modeling and simulation analysis accurately predicted the outcome of the clinical equivalence trials, and clinical equivalence of both FDCs studied was demonstrated. This example demonstrates how modeling approaches can be leveraged in late stage clinical development space to successfully predict the effectiveness of new dosage formulations. Furthermore, such an approach could potentially eliminate the need for dedicated clinical efficacy trials after near-miss BE results in the future, which could lead to reductions in time to market and enable more rapid patient access to more convenient dosing options.

## Cardiovascular safety de-risking

Model-based analyses can be used to de-risk and better understand possible cardiovascular safety concerns for investigational drugs early in development. For example, the QTc interval is routinely investigated in Phase 1 development as a means to determine the propensity to cause cardiac arrhythmias through delayed repolarization. Following the release of the International Conference on Harmonization (ICH) E14 guideline in 2005, regulators required a “thorough QTc” (TQT) study to evaluate whether investigational drugs prolong the QTc interval [19, 20]; however, in 2015, Darpo et al. [21] reported the results of a study that paved the way for ER modeling of Phase 1 data to evaluate the effect of investigational drugs on the QTc interval as a potential alternative to TQT studies. The published study utilized ER methods to evaluate and correctly classify QTc prolongation risk of six drugs, five of which had a known QTc effect and one of which was known not to have a QTc effect, thus validating the use of an ER approach in Phase 1 to inform propensity of investigational drugs to prolong the QTc interval. Furthermore, ICH released a document in December 2015 (“E14 Q&A’s (R3)”) that supported the use of concentration‐QTc (C-QTc) modeling for regulatory decisions [22].

Prior to Darpo et al. [21] carrying out a formal study and presenting the results in a joint regulatory-public forum, various sponsors and academic groups used ER or concentration-QT (C-QT) analyses to de-risk compounds early on in their development, prior to investing in a TQT trial. One such case (for omarigliptin) is presented below, demonstrating the utility of data collected in early phase development to assess the risk of arrhythmogenicity ahead of a TQT trial with comparable conclusions. In the current regulatory environment, robust electrocardiogram (ECG) sampling in early phase studies coupled with ER analyses such as the one presented below, along with extensive pre-clinical assessments of the compounds under the Comprehensive in vitro Proarrhythmia Assay (CiPA) paradigm [23], may be sufficient to be used in place of a TQT study to assess the propensity of novel therapies to cause arrhythmias and could be the basis of discussions with Regulators for a TQT waiver.

### Example: Informing QTc risk early in development

Omarigliptin is a dipeptidyl peptidase-4 inhibitor approved in Japan as a once-weekly treatment for type 2 diabetes. Early in clinical development, an exploratory C-QTc analysis was conducted by Krishna et al. [24] using omarigliptin plasma concentrations and time-matched triplicate 12-lead ECG data from the first-in-human single rising dose study in healthy male subjects. The C-QTc analysis results indicated a linear relationship between omarigliptin concentration and Fridericia-corrected QT (QTcF) interval, with a non-significant slope of 0.2797 ms/μM (95% CI = − 0.2523 to 0.8117 ms/μM). The point estimate of the slope predicted an approximate 2.8 ms prolongation at omarigliptin exposures up to 10 μM (~ 17-fold above the typical clinical maximum concentration [C_max_] of 600 nM in patients), which was well below the threshold of regulatory concern of 10 ms. Therefore, these results suggested a low likelihood of clinically significant QTc prolongation at therapeutic or supratherapeutic doses of omarigliptin and informed risk of drug-induced arrhythmogenicity early in clinical development.

Based on requirement in the ICH E14 Guidance at the time, a definitive TQT trial was later conducted [25]. The results of the TQT study confirmed the first-in-human modeling that a supratherapeutic dose of 175 mg (7 × the clinical dose) did not prolong the QTc interval. This example illustrates how model-based approaches and first-in-human data can be leveraged early in development to inform the risk of drug-induced cardiovascular safety events.

In addition to routine QTc assessments, hemodynamic responses, such as blood pressure (BP) are often monitored in early phase trials. In cases where an unintended effect on hemodynamics (or other safety signal) is observed, ER analyses may be conducted to help inform the therapeutic window and possible development strategies to mitigate the unintended effects. One such example is presented below, in which an elevation in blood pressure was observed in Phase 1 for a compound (compound A) in development for Parkinson’s disease. ER modeling and simulations were used to help guide formulation strategies to ameliorate the BP elevation.

### Example: Informing development for a compound with unexpected blood pressure effects

In a first-in-human trial of compound A, which was in development for Parkinson’s disease, an undesirable transient elevation in BP was observed. This transient elevation was more apparent with increasing dose and was on the order of a few hours at the maximum dose studied. In order to better characterize the BP response, Stroh et al. [26] developed a PK/PD model that incorporated important aspects of mechanisms of BP homeostasis based on the first-in-human trial data. The BP PD model included four main components to characterize the BP response: a sinusoidal BP set point, an effect compartment, a linear effect model, and a system response.

The PD model was coupled with a minimal PK model in order to explore approaches for minimizing the undesirable BP increase, including development of oral controlled-release (CR) formulations. The PK/PD model was used to simulate BP responses for theoretical formulation release rate profiles, and the results suggested some amelioration of the peak BP response with CR formulations. These results supported triggering subsequent CR formulation development, after which actual dissolution data from candidate CR formulations were used in the PK/PD model to confirm a predicted potential benefit in the peak BP response prior to testing in the clinic. Thus, the model-based approach applied for this program to characterize an undesirable cardiovascular adverse event supported decisions on next steps in advancing the program.

## Discussion

In this manuscript, examples of how modeling and simulation approaches have been applied in the development of cardiovascular drugs at various stages of development have been presented. These model-based analyses greatly impacted and informed development decisions and strategies and highlight the utility of applying modeling and simulation strategies throughout development. The examples presented illustrated how such analyses can inform success of a discovery phase program (fIX inhibitor [11]), inform dose selection for Phase 3 trials (anacetrapib [15]), evaluate whether dose adjustments are needed based on intrinsic and extrinsic factors (vorapaxar [16]), and evaluate the clinical relevance of not achieving bioequivalence for an FDC relative to coadministration of individual component drugs (ezetimibe + atorvastatin FDC [17]). Also discussed were examples of how using modeling and simulation approaches in early phase development helped inform strategies to mitigate an unintended cardiovascular-related side effect (BP elevations in the compound A example [26]) as well as informed the propensity of novel therapies to cause cardiac arrhythmias (using QTc as an early indicator in the omarigliptin example [24, 25]). These examples illustrate how modeling and simulation approaches can be leveraged to achieve more informed drug development in the cardiovascular space.

The presented examples leveraged various quantitative approaches, including model-based meta-analysis, population PK and PK/PD modeling, translational modeling, and PK/QTc and PK/AE modeling, all of which were empirical in nature. However, there has been a recent trend in the pharmaceutical industry toward development of quantitative systems pharmacology (QSP) models. QSP models are mechanistic models that describe in detail important factors of the pathophysiology of disease and provide opportunities to explore how drugs can impact this. They provide a framework for integration, extrapolation, and visualization of data, and represent a promising future path for model-informed drug discovery and development for cardiovascular targets, with applications already developed to explore lipoprotein metabolism and kinetics [27, 28], with a focus on HDL modulation [29], and to explore the pathophysiological mechanisms of hypertension and response to antihypertensive therapies, including in salt-sensitive and salt-resistant hypertensive populations [30, 31].

In order for model-based approaches to have a high degree of impact on drug development decisions and strategies, proactive engagement of the project teams before model development begins and continuing engagement throughout the modeling process is critical. In all the examples presented, there was agreement by the project teams to use modeling and simulation approaches to inform decision-making, as well as engagement from the teams in aligning on modeling assumptions and approaches. The models and associated results were ultimately well received by the drug discovery/development teams.

Of the examples presented, in cases where the models were submitted to regulatory agencies, there were varying responses from the agencies. In the case of the vorapaxar submission, the modeling results were well received by regulatory agencies. However, in the case of the ezetimibe + atorvastatin FDC, the model-based translation of the BE results to efficacy results was not deemed to be sufficient to replace dedicated clinical trials, and additional clinical data were requested by the FDA for the FDC doses that did not meet the bioequivalence criteria [17]. Clinical efficacy studies were conducted, with the modeling and simulation results used to inform study design and optimization. Ultimately, the clinical results and the simulation-predicted results closely matched, and, after review of the clinical data, the FDC was approved by the FDA.

Regulatory agencies are increasingly open to model-informed submissions, and intelligent use of modeling tools is likely to be increasingly important for successful regulatory interactions. In the United States, for example, the Prescription Drug User Fee Act (PDUFA) was recently re-authorized, and the FDA has proposed PDUFA VI performance goals and procedures for fiscal years 2018 through 2022, including a specific goal to advance model-informed drug development (MIDD) through development of expertise in this area in FDA staff, public workshops to discuss relevant topics, and a pilot program including additional opportunities to meet with the FDA and discuss MIDD approaches [32]. The FDA’s continuing investment in and commitment to using modeling and simulation approaches to address drug development, regulatory, and therapeutic questions has also been highlighted in public statements by the current FDA commissioner [33].

In summary, this paper has provided examples of the impact of modeling and simulation on drug development strategies and decisions in the cardiovascular area at various stages of the drug development process. The models informed key decisions and strategies in these development programs, such as whether to advance compounds to the clinic, which dose to advance to late phase trials, how to design efficient clinical studies, how to provide appropriate guidance in the product label, and whether formulation strategies could be used to mitigate an unintended adverse event. Looking to the future, there is potential for model-based approaches to have an even greater degree of impact on drug development due to the changing regulatory environment and the development of more integrative QSP disease platform models to compliment more empirical pharmacometric approaches.
